# Changes in the life history traits in *Aedes aegypti* selected for resistance to permethrin and thymol, a spatial repellent

**DOI:** 10.1371/journal.pone.0329776

**Published:** 2025-08-19

**Authors:** Chelsea T. Smartt, Sara Farless, Natalie L. Kendziorski, Tse-Yu Chen, Yuexun Tian, Barry W. Alto, Sandy A. Allan, Cynthia C. Lord

**Affiliations:** 1 Florida Medical Entomology Laboratory, Institute of Food and Agricultural Science, University of Florida, Vero Beach, Florida, United States of America; 2 Agricultural and Veterinary Entomology – United States Department of Agriculture, Center for Medical, Gainesville, Florida, United States of America; Universidade Federal do Rio de Janeiro, BRAZIL

## Abstract

Mosquitoes are both pests and pathogen vectors. Exposure to insecticides for mosquito control may have unintended outcomes on mosquito biology, including evolution of insecticide resistance. Direct lethal effects from exposure to insecticides reduce population size but may also alter phenotypic traits among surviving adult mosquitoes (e.g., size, longevity, biting, susceptibility to pathogen infection), attributable to changes in density-dependence and sub-lethal exposure. Resistance has increased in many populations as a result of traditional contact insecticide mosquito control measures (e.g., pyrethroids). Spatial repellents, including essential oils, are an increasingly important area of mosquito control as spatial repellents deter entry into dwellings, decrease biting, and development of insensitivity to repellents has not been detected. Effects of exposure to these chemicals have not been well studied, however. This study investigates changes in life history and fitness costs in *Aedes aegypti* selected to be resistant to different doses of permethrin and thymol. *Aedes aegypti* selected to be resistant to a contact insecticide (permethrin) and spatial repellent (thymol) generally exhibited reduced adult longevity compared to the unselected (control) group but these differences depended on selection dose, meal type (unfed, blood-fed, sugar-fed) and mating status. Both avidity to blood feed and the number of eggs laid were higher in mosquitoes selected using both doses of thymol and a high dose of permethrin compared to control mosquitoes. Permethrin and thymol selection resulted in shortened and lengthened development times than controls, respectively. As with pyrethroid based chemicals, use of spatial repellents, such as thymol, may impact important life history traits in mosquitoes. Use of these chemicals may alter mosquito population dynamics and biting pressure. Our results support the notion that development of resistance to spatial repellents can have lasting effects on mosquito biology relevant to their ability to transmit pathogens. Testing of insensitivity to spatial repellents should be incorporated into control strategies, since repellency can be a strong selective force influencing reproduction (e.g., acquisition of a blood meal from a host).

## Introduction

Mosquito exposure to insecticides from direct application used by mosquito control, or as non-target effects from household or agricultural use, may have unintended outcomes. Direct lethal effects from exposure to insecticides reduce population size. However, phenotypic traits among surviving adult mosquitoes may be altered, attributable to reductions in density-dependence and alterations in physiological parameters associated with gene expression modifications (e.g., immune responses) from sub-lethal exposure, observed in mosquitoes and other insects [1–6]. Traditional chemical contact insecticide (CIs, chemicals that are toxic to insects upon direct contact) mosquito control measures have increased resistance in mosquito populations. However, the use of contact insecticides is still the primary method for controlling pestiferous and medically important mosquitoes [7]. Vector-borne pathogens continue to be of concern, so to ensure sustainable control of mosquitoes additional control tools, such as spatial repellents, should continue to be developed.

The use of spatial repellents (SRs) is an important area of mosquito control as SRs deter mosquitoes from entering dwellings and protect outside spaces, by providing a barrier of vaporizing chemical, with the goal of decreasing the numbers of mosquitoes entering a home or protected space. This decreases the biting rate on humans from the mosquito population. However, it is known that populations of mosquitoes that fail to be repelled may be resistant, allowing passage through the SR barrier, similar to resistance developed from sub-lethal exposure to CIs [8]. The most widely used spatial repellents are those derived from pyrethroids, however because of the rise in resistance to pyrethroids, the sensitivity to these repellents has been reduced [9]. Consequently, the use of repellents based on plant-derived compounds has become an attractive and viable alternative method [10,11].

Most plants produce compounds that deter attack from phytophagous insects and these volatile compounds may serve as repellents, feeding deterrents, toxins, or growth regulators, including for biting Diptera [12]. The deterrent compounds include 5 major classes of chemicals such as terpenoids, nitrogen compounds, phenolics, protease inhibitors and growth regulators. The response to several of the chemicals by biting insects is suggested to be an evolutionary relic from a plant-feeding ancestor [13]. Studies, both laboratory and field, investigating the use of plants for mosquito control, including leaves and essential oils, revealed several promising plants that are protective and/or repellent to mosquitoes [12,14,15]. There are also studies testing the toxicity of plant compounds and a few plants were shown to have larvicidal properties [16]. Results from these studies led to increasing interest in plant essential oils (EOs) as repellents (on hosts/surfaces and spatial) and as CIs. For example, the plant-derived monoterpene thymol is associated with high levels of landing inhibition for domestic mosquitoes *Ae. aegypti* and *Ae. albopictus* [17,18]. However, little is known about phenotypic changes following exposure (similar to sublethal effects from CIs) or the likelihood of mosquito populations developing resistance or insensitivity to EOs or their major components [19,20].

Terminology used to describe the response to an insecticide or repellent can vary, so for clarity we will use the following terms. Insecticide resistance indicates that the dose lethal to 95% of the population (LD_95_) is above a designated level. The CDC has designated doses signifying resistance in mosquitoes for many common insecticides. Insecticide tolerance indicates that the LD_95_ has increased above a baseline designated as susceptible, but has not yet reached the resistance level. Repellence is when the presence of a chemical causes the insect to move away or stop approaching the treated surface or area, most often in the context of repelling from a blood-meal host or an area that contains hosts. Deterrence is used similarly, but more often in contexts not related to host seeking. Insensitivity to repellants is when an insect does not respond (e.g., is not repelled) to a chemical designed to repel them.

As resistance can have consequences on many aspects of mosquito biology affecting their ability to transmit pathogens (e.g., inhibition and facilitation), it is important to assess the effects of exposure to potential control agents, including CIs and SRs, on mosquito biology and vector competence. Assessing the impact of population-level differences in insecticide resistance and insensitivity to repellents on pathogen susceptibility allows evaluation of effects on the ability of an arthropod to acquire, maintain, and transmit pathogens (vector competence). As these impacts may increase or decrease components of mosquito fitness or vector competence, it is important to assess multiple traits in the same populations to estimate overall effects on a parameter or index of interest to mosquito control and public health agencies (e.g., mosquito biting pressure and vectorial capacity).

This study describes for the first time the influence of selection against thymol, a thyme essential oil major component, on *Ae. aegypti* life history traits. Changes in longevity, fecundity, adult development, and biting rate are compared among *Ae. aegypti* populations that were selected to be resistant to sublethal doses of permethrin and those selected against doses of thymol. Comparisons were made relative to a genetically similar population of *Ae. aegypti*, without selection to be resistant to permethrin and thymol (unselected control). Selection-influenced changes in the traits and impact on mosquito biology are discussed.

## Materials and methods

### Mosquito collection, rearing and permethrin susceptibility screening

For results relevant to field situations, we required a starting population that was susceptible to permethrin, but also low-passage and genetically similar to field populations. High-passage lab strains are susceptible but may not be genetically similar to current field populations. To achieve this, our starting populations were a Florida field resistant population and a low-passage, susceptible lab colony from the same county.

The field population was collected via oviposition cups containing hay infusion and germination paper set up by employees of Manatee County Mosquito Control District to collect F1 *Ae. aegypti* mosquito eggs in Manatee County, FL (GPS coordinates 27.45982, −82.66264). Egg papers were collected, allowed to dry and sent to the Florida Medical Entomology Laboratory (FMEL) in Vero Beach, FL. This was a component of their regular surveillance, and permits for egg collection were not required. Eggs were vacuum-hatched, and immature stages reared under standardized conditions [21]. Specifically, larvae were separated into pans at an approximate density of 200 larvae per pan (14” W x 17” L x 2.5” D, US Plastics #52054) in 2L water, and provided with 20mL of larval food (5g brewer’s yeast and 5g liver powder mixed with 1L water) daily. Adults were provided 5% sucrose solution-soaked cotton rolls ad libitum. Mosquito colonies were maintained by feeding female mosquitoes on blood from chickens following approved standard protocols (IACUC protocol 201807682) [22]. Adults were briefly anesthetized at 4°C then identified to species and *Ae. aegypti* individuals were used to start a lab colony, designated Manatee F1. In addition to the Manatee F1 population, an F4 *Ae. aegypti* population, originally collected in Manatee County (GPS coordinates 27.459833, −82.662639), was also used in this study. These standard colony rearing methods were used to produce mosquitoes for assays, selection and experiments. Mosquito rearing was performed in a climate-controlled insectary (28°C, 60–80% relative humidity under a light:dark cycle of 14:10 hours) at the FMEL.

CDC bottle bioassays were used to determine susceptibility to permethrin [23]. Permethrin powder (99.5% pure, Chem Service Inc., West Chester PA, US) was dissolved in acetone and 1mL of the diagnostic dose (43 µg/bottle) was used to coat the interior surface of 250mL Wheaton bottles (Thermo Fisher Scientific, Waltham, MA) following the CDC bottle bioassay protocol [23]. Twenty female mosquitoes from each Manatee County, FL population (F1 and F4) were added to the coated bottles and exposed to permethrin. This treatment was repeated four times (four treated bottles) for each population (80 mosquitoes/population). Mosquito survival was monitored every 5 minutes for the first 15 minutes, then every 15 minutes for 2 hours. A bottle coated with acetone only was used as a control and contained the same number of mosquitoes. The F1 Manatee County *Ae. aegypti* population was shown to be resistant to the diagnostic permethrin dose and time while the F4 population was susceptible at the same dose/time. The next generation of female *Ae. aegypti* from the susceptible Manatee lab population (F5) was backcrossed with the males from the resistant field population (F1) to generate a population that had a genetic background more similar to the field population and was still susceptible to permethrin (S1 Fig). The eggs from the new back-crossed *Ae. aegypti* population (Fx2) were obtained. Adults from Fx2 population were evaluated using CDC bottle bioassay as described above and shown to be susceptible to the diagnostic dose of permethrin [23]. Another backcross to increase permethrin susceptibility in the population was made with the Fx2 population males and females from the permethrin-susceptible population from Manatee County (F5). The second generation of this cross is designated Fxx2 and used to select permethrin-resistant and thymol-resistant populations.

### Selection of resistance

Permethrin resistance selections were carried out in a series of dosing experiments in an ascending dose order using 1L Kimble glass bottles with screw caps (Thermo Fisher Scientific, Waltham, MA). Permethrin powder was dissolved in acetone to generate a stock solution of the appropriate concentration, then 1 mL of solution was pipetted into each bottle (4 treatment replicates) and the bottle rolled to coat the interior following CDC Bottle Bioassay Protocol. Thus, each replicate bottle received an equivalent amount of permethrin following CDC Bottle Bioassay Protocol [2,23]. Using the described bottle bioassay method and a range of doses, we determined that Fxx2 population had an LD_50_ of 15 µg of permethrin in this assay. Thus, four permethrin doses in ascending order from that LD_50_ (15 µg, 30 µg, 60 µg, and 120 µg) were chosen for the selection process against five consecutive generations of virgin male and female mosquitoes (S1 Fig). For selection, fifty 7-day-old unmated male and female Fxx2/Fxx3 *Ae. aegypti* were transferred by aspiration into separate bottles and the trial start and stop times were recorded. When 30% of the mosquitoes in each bottle were knocked down, all mosquitoes from treated bottles were collected and placed into screened cages (W24.5 x D24.5 x H24.5 cm) (Bug Dorm, MegaView Science Co., Ltd., Taiwan), provided 5% sucrose on cotton wicks, and returned to standard conditions. The male and female mosquitoes that survived 24-hours post-exposure were allowed to mate and females blood fed on chickens under the same animal protocol. The progeny of these experimental Fxx3 mosquitoes were reared and used for the next generation. Each new generation was exposed to the next dose of permethrin until they also reached a level of 30% mortality from that exposure. Selection continued through five generations. The testing of these doses was carried out to determine sublethal doses and the subsequent impacts of these doses on female mosquito life history characteristics. The *Ae. aegypti* selected for resistance at 60 and 120 µg of permethrin, as described, were then used in subsequent life history trait experiments (S2 Fig).

The thymol selection experiments were performed via an original I-shaped, dual choice chamber olfactometer device consisting of 3 cylindrical acrylic chambers and metal gates to isolate the chambers [24] (S3 Fig). Briefly, the olfactometer consisted of a glass central release chamber with a small portal to insert insects and two side chambers connected with metal shuttered gates. One of the end chambers was designated as the control chamber and the opposite one served as the treatment chamber. Pre-treated cotton pads (Cliganic, San Francisco, US) were placed in the control chamber (acetone only) and treatment chamber (acetone + thymol) and volatile fumes allowed to spread through the testing chamber. Thymol dosing was done by dissolving an appropriate amount of thymol crystals (>98.5%, Sigma-Aldrich Co., St. Louis, Missouri, US) in acetone for the desired mg/mL dose, then 1mL of the solution (treatment) or acetone (control) was pipetted onto each cotton pad. The device was used to determine the thymol dose with the lowest (1.2 mg) and highest (3.0 mg) repellency for the same Fxx2/Fxx3 mosquitoes then two mid-range concentrations were picked to yield four thymol concentrations to test (1.2, 1.7, 2.1, 3.0 mg). Six replicate treatment and control cotton pads were prepared, then allowed to sit for 30 minutes for acetone to evaporate before use in the chambers. The cotton pads contained either control (acetone) or test (thymol) concentrations (1.2, 1.7, 2.1, 3.0 mg) and were used in the selection process against five consecutive generations of virgin male and female mosquitoes (S1 Fig). This range finding preliminary study showed increased mortality in males over concentrations of 2 mg and for females over 3 mg.

Twenty-five 7-day old unmated females from the Fxx4 permethrin-susceptible *Ae. aegypti* population (S1 Fig) were retrieved from a colony cage containing only females using a draw box to capture female mosquitoes willing to fly [25]. Twenty-five males were retrieved from a colony cage of the same population, containing male and female mosquitoes. The retrieved males and females were aspirated into the central release chamber through the access portal in the middle and allowed to acclimate while control and treatment doses were prepared. The treatment and acetone (control) treated cotton pads were added to opposite ends of the chambers and the assay initiated by opening the gates on both sides of the central chamber and allowing mosquitoes to move throughout the device for 10 minutes. After 10 minutes the gates are closed and mosquitoes from each chamber recovered into clean cages corresponding to chamber type (i.e., treatment and control), counted, and provided 5% sucrose solution. The males and females from the treatment chambers were allowed to mate and blood fed from chickens (IACUC Protocol above). The progeny of these thymol-exposed and resistant mosquitoes were reared to be used in repeat selections. *Aedes aegypti* selected through five generations at two thymol doses, 2.1 and 3.0 mg, were used in the life history trait experiments (S2 Fig). These doses were chosen for the life history experiments based on observable repellence and lower mortality in females during selection.

### Life history traits estimated for permethrin (CI) and thymol (SR) resistant populations

Life history traits were obtained from Fxx4 *Ae. aegypti* progeny, selected at 2 doses of permethrin (60, 120 µg) and thymol (2.1, 3.0 mg) (S2 Fig, sample sizes in S1 Table). These were hatched and reared under standard colony conditions. Pupae from the selected populations and the unselected control population of similar generation were sorted by size in plastic containers with water, where larger female pupae can be separated from smaller male pupae [26] then placed into screened cages of males only, females only or mixed. The female only groups were used for testing longevity of unmated females and mixed sex groups used to test longevity of mated females. Up to 150 adult unmated and mated females were placed into separate cartons (16 oz food cups, WebstaurantStore, Lancaster PA) with mesh screen (Joann Fabric, Hudson, OH) secured to the top with a rubber band, provided 5% sucrose solution on cotton wicks, and adult mortality monitored daily to obtain longevity data of the females. Final sample size per carton ranged from 39–55 depending on available adults from the same rearing cohort. The remaining mated females were used for longevity of bloodfed mated females, biting rate and fecundity experiments (S2 Fig). Approximately 700 adult females/treatment group (= mixed sex (mated) or female only (unmated) x 5 populations) were provided commercially available bovine blood (Hemostat Laboratories, Dixon CA), supplemented with ATP (1mM) as its ingestion supports mosquito biology [27], using a Hemotek blood feeding system (Hemotek, Blackburn, UK). Fully engorged females were sorted and up to 200 per treatment were placed in screen topped cartons (16 oz, WebstaurantStore), provided 5% sucrose solution on cotton wicks, and longevity post blood meal monitored daily. Final sample size depended on feeding success and ranged from 3 to 43 females/carton with six cartons per population (S1 Table). From the remaining blood-fed females, up to 200 females per treatment were placed individually in DWK Life Sciences Wheaton vials each containing an oviposition paper and water (Thermo Fisher Scientific, Waltham, MA). Vials were monitored for eggs and eggs were counted at 5–8 days post blood feeding. Following egg counts, egg papers were placed in small cups with tap water and allowed to hatch for 2 days before first instar larvae (L1) were counted.

Biting rate estimates were performed with 200 mated females from each treatment group. Four cartons with 50 females/treatment were provided the opportunity for a blood meal every day for 5 days. Blood-fed mosquitoes were sorted from non-fed individuals, and both placed into separate cartons after each new blood meal. Magnification (10x) and a light source were used to determine if fed females had taken a blood meal (fresh blood looks red inside the midgut). The number of blood-fed and non-fed mosquitoes were counted each day.

For longevity, the final treatment groups were blood-fed and mated, not blood-fed and unmated, not blood-fed and mated, across the 5 populations. For fecundity and biting rate, final treatment groups were the populations. The parental population (unselected) used for the backcross experiments is designated as parental. The control unselected group, subsequent generations from the second backcross, is designated as Fxx4/5 or control. Fxx4 was used as control for most studies.

An additional generation was needed to produce eggs in the experiment assessing development time of thymol selected mosquitoes, so generation Fxx5 was used. Selected populations are designated by the chemical and selection dose (permethrin, 60 and 120 µg; thymol, 2.1 and 3.0 mg). The development time study used mosquito eggs from the same generation and were vacuum hatched. Standard rearing conditions were used, except the number of larvae per pan was counted rather than approximated. Two hundred L1 were added to each rearing tray with 3-fold replication per treatment group. However, due to the small size of L1 there was still some variation in the exact number of L1 per tray. When pupae began to develop, the number of pupae per tray was counted daily. The counted pupae were added to pupal cups and placed in screened cages per treatment/day of emergence and provided a 5% sucrose solution. Emergence of adult males and females was recorded each day.

### Statistical analyses

Adult longevity of *Ae. aegypti* were analyzed using survival analysis by fitting the survival time to the exponential model to obtain the hazard function (λ). Model selection was performed on the hazard function to evaluate the effects of 3 factors on adult longevity: population selection regime (P: chemical x selection dose or unselected control); mating status (M: mated or unmated); and blood feeding status (F: blood fed or sugar fed). Linear regression models were fit for the 3-factor model, three 2-factor models, and the three single-factor models (8 total) without including interactions, and AIC_c_ used to assess fit. These analyses were done separately for each chemical, to assess if different models best fit the data. Summary statistics (mean, median, SE) were calculated for survival to provide additional information and easier interpretation of effect on lifespan. Model selection analysis incorporates variation in the data, so additional variance analyses would be redundant and were not done.

In the selection of resistance on biting rate study, the Kruskal-Wallis test was used to compare the number of individuals that fed from each population, followed by Tukey-Kramer means comparisons. For fecundity, one-way ANOVA and Tukey’s multiple comparison test were used to compare populations in the selection of resistance for the number of eggs laid by females ovipositing, while *Χ*^2^ tests were used to compare the proportion of females that oviposited and the proportion of clutches that hatched. Significance was adjusted for multiple tests using the Bonferroni correction. In the selection of resistance on development to adult study, both development time from egg to adult and gender of each individual mosquito were recorded. Development time from first instar to adult (by sex) was averaged by replicate trays, then tray means used for one-way ANOVA followed by Tukey’s multiple comparisons test. Kruskal-Wallis analyses, followed by Tukey-Kramer means comparisons, were used to compare the proportion of pupae successfully eclosing (# eclosed adults/# pupae), and proportion female (# females/# adults eclosing) across selected and unselected lines within chemicals were analyzed using *Χ*^2^ tests. Because these experiments had to be done using different generations for the two chemicals, chemicals could not be directly compared. Survival to pupation was not analyzed as there was low but unmeasurable variation in the number of first instar larvae in each tray. The biting rate analysis, survival analysis and model selection were performed using Matlab (versions 2021a and 2020b, MathWorks, Portola Valley, California, USA); fecundity and development analyses were performed in Prism (version 10.3.1, GraphPad Software, Boston, Massachusetts, USA). Proportion female and proportion of egg batches hatching (*Χ*^2^ tests) were performed in SAS (version 9.4, SAS Institute, Carey, North Carolina, USA).

## Results

### Effects of selection for resistance on longevity

**Fig 1:**
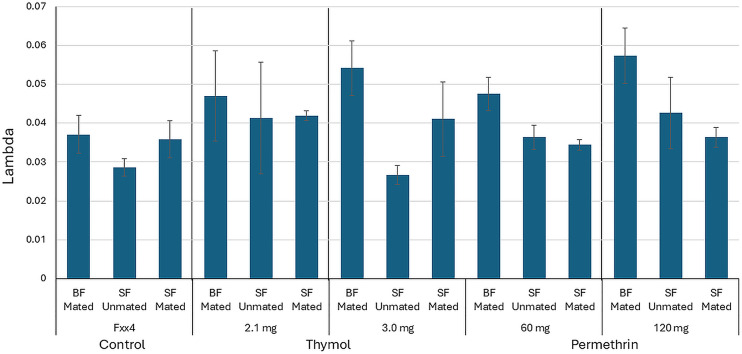
Estimated daily mortality rate (lambda, λ) for control population (Fxx4) and selected populations. Model selection indicated that all 3 factors (population, blood feeding, mating) affect the mortality rate. BF = Blood-fed, SF = Sugar-fed.

The estimated daily mortality rate from the fitted exponential models, λ, varied between populations, mating and feeding status (blood or sugar) (Fig 1). Higher rates were generally observed in blood-fed, mated females. The contribution of each factor (population selection regime P, mating status M, blood feeding status F) to the mortality rate was explored using model selection. Feeding status and mating status are separate variables to isolate the contribution of the factors. However, as females will not blood feed until mated, the combination sugar-fed and mated could not be done. See methods and Table 2 for details. For permethrin, the models with all 3 factors and the 2-factor model with feeding status and population were the best descriptions of the data (Table 1), with other 2-factor and single factor models not well supported (ΔAIC_c_ > 6). The best supported models had R^2^ > 0.7. For thymol, three models were well supported, the 3-factor model and 2-factor models including population and feeding status and population and mating status (Table 1). However, R^2^ was lower for the best thymol models than for the best permethrin models, ranging from 0.39 to 0.44. The remaining 2-factor model (feeding status and mating status) and single factor models had less support (ΔAIC_c_ > 4) and were not as good descriptions of the data. The population selection regime was consistently in the best models, for both permethrin and thymol selection. For both chemicals, there was unexplained variance indicating that other factors are influencing the mortality rate, more so for thymol. These factors could include levels of sugar feeding and size of blood meals, which were not measured, or individual genetic variation. However, all 3 factors played a role in longevity, with mating and blood feeding increasing the mortality rate relative to the sugar only, unmated treatment.

**Table 1:** Model selection results with feeding status, mating status and population as independent variables for mortality rate.

Model^a^	Permethrin			Thymol		
	AICc	ΔAIC_c_^b^	R^2^	AICc	ΔAIC_c_^b^	R^2^
**F + M + P**	−175.72	0.97	0.74	−233.33	**0.00**	0.45
**F + P**	−176.69	**0.00**	0.71	−232.39	0.94	0.39
**M + P**	−160.84	15.85	0.44	−232.38	0.95	0.39
**F + M**	−169.23	7.46	0.61	−228.87	4.46	0.28
**F**	−170.54	6.15	0.59	−228.44	4.89	0.22
**M**	−158.54	18.16	0.32	−228.43	4.90	0.22
**P**	−152.62	24.08	0.13	−223.85	9.48	0.17

^a^F = feeding status (levels: blood-fed, sugar-fed only); M = mating status (levels: mated, unmated); P = population (levels: Control Fxx4, chemical and selection dose (permethrin 60 or 120 µg; thymol 2.1 or 3.0 mg).

^b^Model with the lowest AIC_c_ (ΔAICc = 0) for each chemical is bolded.

**Table 2:** Median and mean ± SE survival time (days) for the 5 populations and combinations of feeding and mating status.

Populations	Sugar-fed & unmated		Sugar-fed & mated		Blood-fed & mated	
	Median	Mean ± SE	Median	Mean ± SE	Median	Mean ± SE
Fxx4 (control)	37	35.07 ± 2.86	31	28.23 ± 3.64	29	27.4 ± 3.86
Thymol 2.1 mg	26	26.61 ± 10.50	24	23.87 ± 0.74	21	22.63 ± 6.46
Thymol 3 mg	39	37.75 ± 3.40	27	25.36 ± 6.39	19	18.73 ± 2.21
Permethrin 60 µg	29	29.11 ± 1.17	29	27.65 ± 2.45	20	20.42 ± 1.94
Permethrin 120 µg	28	27.62 ± 1.90	26	22.98 ± 4.66	20	18.11 ± 2.27

Longevity estimates across replicates by population for selected *Ae. aegypti* populations and Fxx4 parental control *Ae. aegypti*.

**Fig 2:**
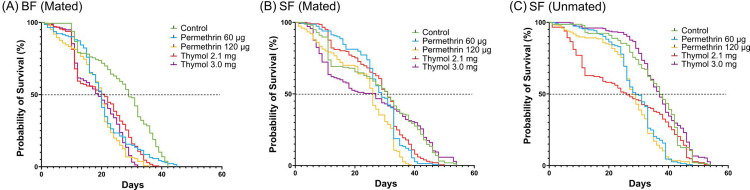
Survival time of permethrin resistant and thymol selected *Ae. aegypti* after mosquitoes were fed: A) BF (Mated); B) SF (Mated); C) SF (Unmated). BF = Blood-fed, SF = Sugar-fed, Control = Fxx4 population, % = percent, µg = microgram, mg = milligram.

For easier interpretation, survival times are also shown in Fig 2 and Table 2, and the same patterns are apparent. Blood feeding reduced average survival time relative to sugar-fed only, across all populations. Median survival time ranged from 39 days (sugar-fed only) to 19 days (blood-fed), with the extremes from the thymol 3.0 mg selection population, suggesting interactions between population and feeding status (Table 2). Differences among the populations are more notable for blood-fed and sugar-fed only, whereas the differences are less notable for unmated mosquitoes. Further analysis was not conducted as it would be redundant to the model selection analysis on the daily mortality rate, λ. Age-dependent mortality may be a factor, as most thymol selected populations showed increased mortality among young mosquitoes (under 10 days old). For individuals only fed sugar, mated females showed the least separation of the populations (Fig 2).

### Selection of resistance on biting rate

**Fig 3:**
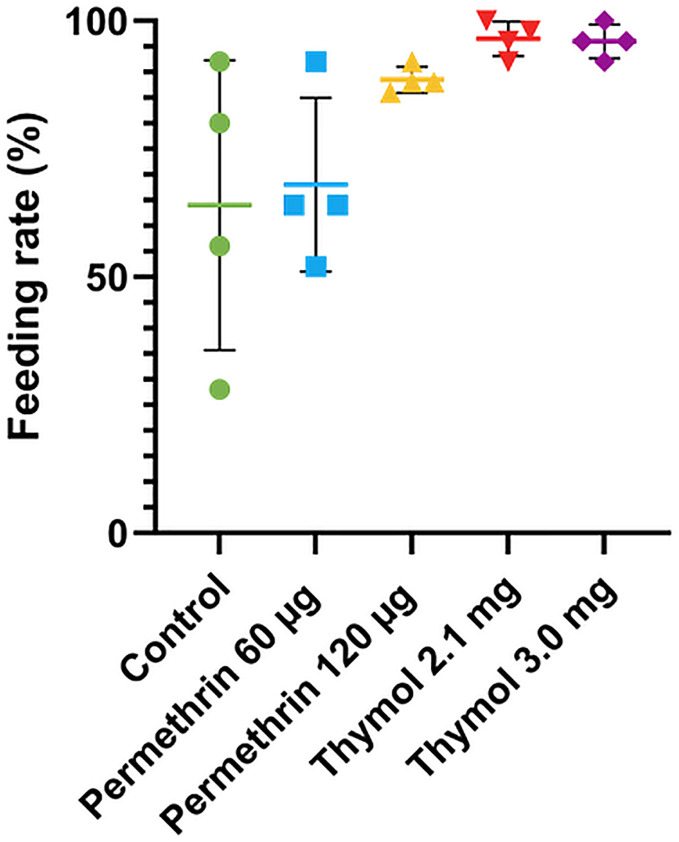
Feeding rate of control, permethrin resistant and thymol selected *Ae. aegypti* populations for the first blood meal of the biting rate assessment. Control = Fxx4 population, % = percent, µg = microgram, mg = milligram. Green circle = Control Fxx4; blue square = Permethrin 60 µg; yellow up triangle = Permethrin 120 µg; red down triangle = Thymol 2.1 mg; purple diamond = Thymol 3.0 mg. Symbols show the feeding rate for each replicate, lines are mean ± SE percent feeding for treatment groups and control Fxx4. No significant pairwise differences were observed (Kruskal-Wallis test, see text and Table 3 for details).

We report here the results for the first blood meal only, a more complete analysis of repeated feeding will be presented elsewhere. The number of females feeding varied between populations (Fig 3, Table 3). The variance between cartons was notably higher in the population selected with permethrin 60 µg and the control Fxx4 population, such that while the Kruskal-Wallis test showed differences between populations (Table 3), the multiple comparisons tests did not detect pairwise differences between groups (S2 Table). The source of variation between cartons within the two treatments with high variance is unknown, but the decreased variation in other populations may be related to the selection process. The comparison showing the strongest difference was between selected with thymol at 2.1 mg and control Fxx4 (p = 0.066, other comparisons p > 0.07, S2 Table).

**Table 3:** Kruskal-Wallis table for proportion blood feeding.

Source	SS	df	MS	*Χ* ^2^	Prob> *Χ*^2^
**Population**	452.875	4	113.218	13.207	0.010
**Error**	198.625	15	13.241		
**Total**	651.5	19			

### Selection of resistance on fecundity

**Fig 4:**
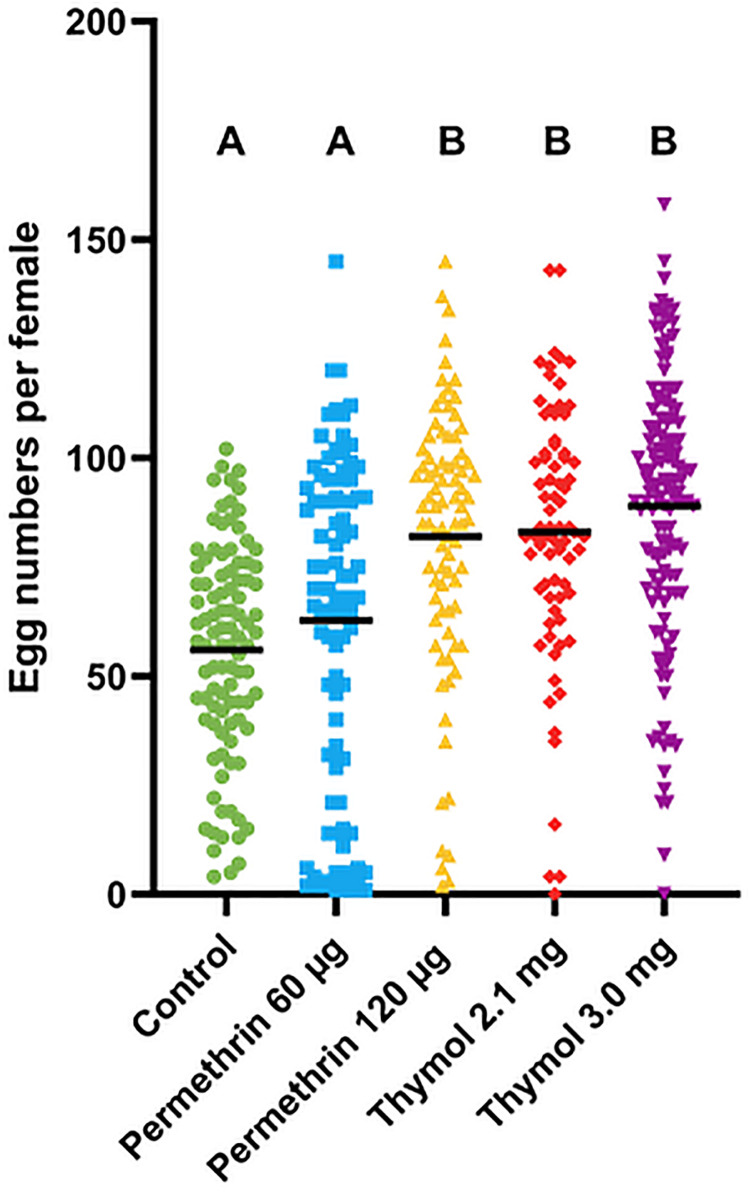
Fecundity of individual blood-fed permethrin and thymol selected female *Ae. aegypti* mosquitoes (with number of females laying zero eggs removed). ANOVA followed by Tukey’s multiple comparison test; letters indicate significant differences between treatments. Control = Fxx4 population, µg = microgram, mg = milligram. Green circle = Control; blue square = Permethrin 60 µg; yellow up triangle = Permethrin 120 µg; red down triangle = Thymol 2.1 mg; purple diamond = Thymol 3.0 mg.

The treatment groups were significantly different in number of eggs (egg “batch”) laid by females that laid >0 eggs (ANOVA F = 23.45, DF 4, 469, p < 0.0001, S3A Table). All selected lines except permethrin 60 μg laid more eggs than the control Fxx4 mosquitoes (Fig 4, S3B Table). Populations varied in the proportion of females that laid eggs (*Χ* ^2^ (1,4) = 34.59, p < 0.0001, pairwise *Χ* ^2^ comparisons in S3C Table). *Ae. aegypti* females selected with the high dose of permethrin were less likely to lay eggs, while neither selected population differed significantly from the control Fxx4 in the proportion of mosquitoes that laid eggs (Table 4, S3C Table). Hatch rate was defined as the proportion of individual female egg batches where any L1 were observed in the vials following flooding. In some cases, we observed eggs that appeared to have hatched, but no L1 were present; these batches were counted as unhatched. The presence of L1indicated that at least some eggs in the egg batch hatched, but accurate counting was difficult so the number of L1 was not analyzed further.

**Table 4:** Effect of selection with permethrin and thymol on fecundity and egg hatching for the *Ae. aegypti* populations.

Population	Total # of eggs laid	Number of females tested	Number of females not laying eggs	Females laying eggs (prop)^b^	Total # L1 larvae	Hatched egg batches (prop)^a,b^
**Fxx4**	5530	174	76	0.56 ab	1098	0.73 ab
**Permethrin 60 µg**	5520	126	38	0.70 b	990	0.55 a
**Permethrin 120 µg**	6868	177	94	0.47 a	2020	0.80 bc
**Thymol 2.1 mg**	6279	167	92	0.45 a	2766	0.96 d
**Thymol 3.0 mg**	11,652	191	61	0.68 b	3009	0.83 bcd

^a^Individual female egg batches scored as hatched if any eggs hatched (L1 observed), unhatched if no larvae were produced.

^b^Females laying eggs and hatched egg batches: proportions followed by the same letter are not significantly different (*Χ*^2^ tests: S3 and S4 Tables).

The proportion of egg batches that hatched varied significantly across all treatments (*Χ* ^2^ (1,4) = 36.10, p < 0.0001, pairwise *Χ* ^2^ comparisons in S3D Table). Selection at the 60 µg permethrin dose negatively impacted hatch rate compared to 120 µg population, and both permethrin-selected populations had lower proportion hatching than the lower dose of thymol (Table 4, S3D Table). Mosquitoes selected for resistance to thymol at both doses laid significantly more eggs than the control population (Fig 4, S3B Table). Females selected at 60 µg permethrin laid fewer eggs than those selected with 120 µg permethrin or either dose of thymol (S3B Table). Additionally, selection with 3.0 mg of thymol yielded a higher percentage of female mosquitoes that laid eggs (68%) compared to those selected at 2.1 mg (44.9%) (Table 4, S3C Table) while for permethrin the lower dose had a higher percent of females that oviposited (69.8% vs. 47%). Thus, selection impacted fecundity for both chemicals but in different ways. However, significantly more egg batches laid by mosquitoes selected with 2.1 mg of thymol (96%) hatched compared to control Fxx4 (73.5%) or either dose of permethrin (Table 4, S3D Table).

### Selection of resistance on development to adult stage

Development time, proportion eclosing, and sex ratios of adults were assessed for all groups. Percent pupating is reported in Table 5 for reference but was not analyzed as the initial number of larvae per pan varied slightly around the target of 200 L1. Permethrin and thymol populations were analyzed separately as these experiments used different generations of mosquitoes. For permethrin selected *Ae. aegypti* mosquitoes, development success of larvae to pupae was high for both treatment groups although lower for mosquitoes selected at 120 µg permethrin. The proportion of pupae eclosing as adults was highest for the control Fxx4 population and lower for the permethrin selected populations, but the differences were marginally nonsignificant (Kruskal-Wallis test, p = 0.0793, S5A Table, Table 5). Proportion of eclosed adults that were female did not differ significantly between permethrin treatments and Fxx4 control populations (Kruskal-Wallis test, p = 0.2881, S6A Table, Table 5). Thymol treatment groups were significantly different for the proportion eclosing (Kruskal-Wallis test, p = 0.0273, S5B Table, Table 5), with both selected populations having higher proportion eclosing than the control Fxx5 population (S5C Table, Table 5). Differences between populations for proportion female were marginally non-significant (Kruskal-Wallis test, p = 0.058, S6B Table, Table 5) with the population selected at 3.0 mg thymol having the highest proportion females.

**Table 5:** Development success: pupation, eclosion (proportion) and numbers of each sex (mean ± st. dev over replicate trays), sex ratio (proportion female).

Population^a^	Pupated (n)	Pupated (%)	Female (n)	Male (n)	Eclosed (prop)^b^	Female (prop)^c^
**Fxx4**	195.7 ± 4.2	97.8 ± 2.1	89.7 ± 2.1	97.3 ± 5.0	0.96 ± 0.01	0.48 ± 0.02
**Permethrin 60 µg**	188 ± 1.7	94 ± 0.9	89.7 ± 5.1	85.7 ± 4.5	0.93 ± 0.02	0.51 ± 0.03
**Permethrin 120 µg**	184.3 ± 3.2	92.2 ± 1.6	79 ± 13.2	100.7 ± 12.1	0.97 ± 0.01	0.44 ± 0.07
**Fxx5**	194 ± 0	97 ± 0	86.7 ± 1.2	96.7 ± 4.5	0.95 ± 0.02 a	0.47 ± 0.01
**Thymol 2.1 mg**	197.7 ± 2.1	98.8 ± 1.4	97 ± 3.5	95.3 ± 10.8	0.97 ± 0.00 b	0.50 ± 0.02
**Thymol 3.0 mg**	196.7 ± 5.1	98.3 ± 2.4	102 ± 3.6	87.7 ± 8.1	0.97 ± 0.00 b	0.54 ± 0.03

^a^Fxx4 and Fxx5 represent the control populations. Due to the time needed for selection and life history trait evaluation, an additional generation of control mosquitoes was necessary for egg production. Development time was evaluated simultaneously for Fxx4 and permethrin-selected populations and for Fxx5 and thymol-selected populations. Pans started with ~200L, 3 replicate pans/treatment.

^b^Eclosion is the number of adults emerging/number of pupae per tray. For thymol-selected and Fxx5 populations, means followed by the same letter are not significantly different. Proportion eclosed for thymol: proportions followed by the same letter are not significantly different. ^c^Kruskal-Wallis tests were not significant for proportion female, either chemical, or proportion eclosed for permethrin. Means comparisons not reported when the primary test was not significant.

**Fig 5:**
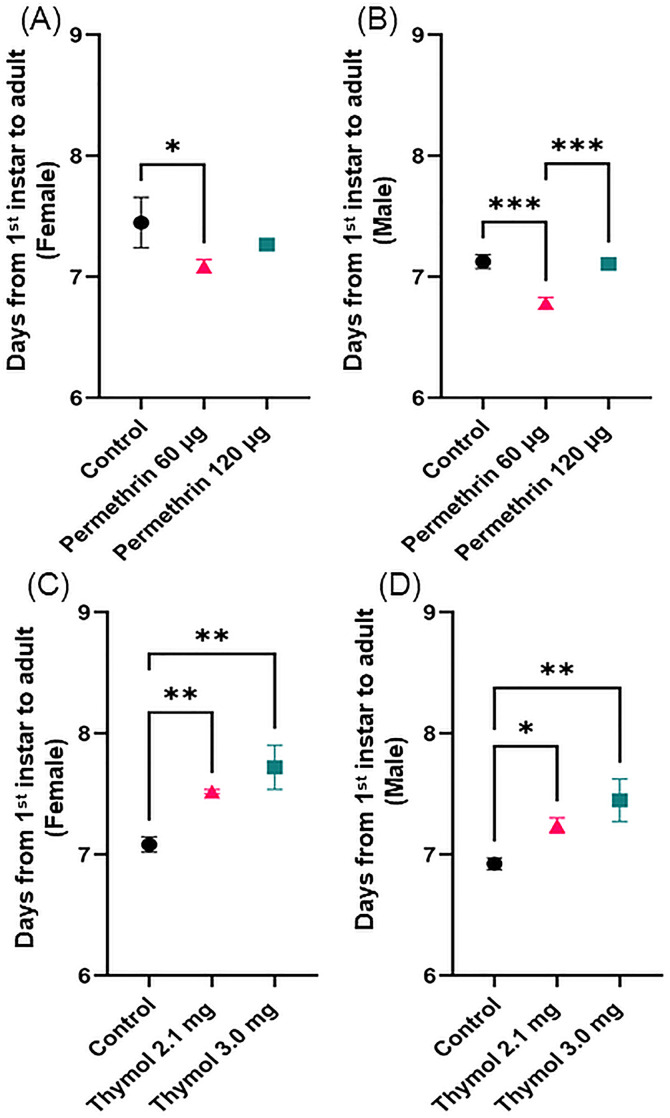
Development (days) from L1 to adult for permethrin resistant and thymol selected female and male *Ae. aegypti* mosquitoes. A) Development time for permethrin resistant female *Ae. aegypti*; B) Development time for permethrin resistant male *Ae. aegypti*; control = Fxx4 population; C) Development time for thymol selected female *Ae. aegypti*; D) Development time for thymol selected male *Ae. aegypti*. Control = Fxx5 population (the number of Fxx4 eggs were insufficient so we used the next generation). The asterisks (*) represent significant pairwise differences; p-values given in S4 Table. µg = microgram, mg = milligram. Black circle = Control; red triangle = low dose; teal square = high dose.

Overall, selection at different doses influenced the time for immature development until adult eclosion for both chemicals and both sexes, although the relationships were different (Fig 5, Table 6, S4 Table). Male *Ae. aegypti* selected to be resistant to the high dose of permethrin had similar development time (7.1 days) as control Fxx4 male mosquitoes. However, males selected at the lower dose of permethrin developed significantly faster than the other two groups (6.8 days, Fig 5, S4A, B Table). In female *Ae. aegypti*, selection with permethrin decreased development time but only the comparison between control Fxx4 and the lower dose was significant (Fig 5, Table 6, S4C Table). Selection with the two chemicals resulted in different patterns of development time between populations. Thymol selection at both doses resulted in longer development times for both male (>6.9 days, S4D, E Table) and female *Ae. aegypti* (>7.0 days, Table 6, S4F Table) compared to the control Fxx5 mosquitoes (Fig 5), although development times were not significantly different between thymol selection doses. Selection with 3.0 mg of thymol resulted in the longest development times observed.

**Table 6:** ANOVA table for adult female development (days from L1 to adult).

Source columns	SS	DF	MS	F (DFn, DFd)	p-value
Permethrin					
Treatment	0.1945	2	0.09724	F (2, 6) = 5.991	**0.0371**
Residual	0.09738	6	0.01623		
Total	0.2919	8			
Thymol					
Treatment	0.6394	2	0.3197	F (2, 6) = 25.35	**0.0012**
Residual	0.07565	6	0.01261		
Total	0.715	8			

Permethrin and thymol treatments analyzed separately, with their respective control populations (Fxx4, Fxx5). Treatment (control, high or low dose) was significant for both chemicals.

## Discussion

We were interested in characterizing changes in those mosquito life history traits that matter the most for parameters in vectorial capacity, resulting from the development of resistance following exposure to sublethal doses of permethrin and thymol, used as a spatial repellent. A parental *Ae. aegypti* population served as the common ancestor for establishing populations selected to be resistant to a high and medium dose of two insecticides with different modes of action. Following the selection regime, the subsequent generations of the ancestral population (control) and insecticide/repellent selected populations were compared to gauge longevity, fecundity, biting rate and development to adulthood.

### Longevity

Permethrin. Adult females from the control population generally lived longer (lower λ) than any of the selected populations, and this was most noticeable in mated mosquitoes (blood or sugar fed). These results support the notion that selection pressure imparts fitness costs likely affecting physiological functions. Other studies showed resistance to permethrin shortens life span in female *Ae. aegypti* compared to the control population [28,29]. However, Chen et al. [2] revealed that an *Ae. aegypti* population selected to be highly resistant to permethrin (LD_50_ = 400 µg) had significantly longer survival than the parental mosquitoes. The differences in longevity in the mosquitoes from these studies may be because Chen et al. [2] selected for resistance using a higher permethrin dose while the current study investigated changes due to resistance to sublethal doses, suggesting that changes in mosquito life span may be influenced by dose and repeated exposure. This suggests that repeated exposure to insecticides over generations can result in consequences for altered life histories of the surviving progeny. The influence of selection to permethrin on estimated daily mortality rate and average longevity was influenced by meal type. Specifically, blood-fed selected females were associated with shortened survival (higher mortality rate λ) which is notable given the wide use of pyrethroids in mosquito control, and these mosquitoes pose the greatest risk for transmission of arboviruses relative to sugar-fed females. The impact of blood on decreasing life span of deltamethrin resistant mosquitoes has been described for *Anopheles funestus,* and the effects increased with additional exposure to the chemical [30]. Collectively, these observations suggest that pyrethroid resistance is often associated with a trade-off in survival of adult mosquitoes, but that other outcomes are also possible.

Thymol. Selection with thymol reduced life span with a noticeable dose response, and blood feeding increased mortality in thymol selected *Ae. aegypti*. Shortened longevity could be due to the toxic nature of thymol. In fact, Padney et al. [14] compared the repellency and toxicity of essential oils extracted from the seeds of the *Trachyspermum ammi* plant with its major purified constituent, thymol, on *An. stephensi*. The study found that thymol was more toxic to adults and more repellent to *An. stephensi* compared to the essential oils. Similar effects of shortening of adult lifespan following thymol exposure have been observed in unrelated insects which may suggest a conserved physiological response among at least some insect taxa (bean weevil *Acanthoscelides obtectus* [31]. From these studies, it can be concluded that thymol selection and exposure impact the adult lifespan of female mosquitoes.

### Biting rate

Determination of avidity of the selected populations in taking an initial blood meal was analyzed as part of the biting rate estimation. Fewer mosquitoes selected at the 60 µg dose of permethrin ingested blood compared to those selected at the high dose. Oliver et al. [30] showed that sensitivity to permethrin decreases with age and blood feeding, especially following multiple meals [28,32]. Consistent with our observations, a 6-fold increase in biting rates was observed in phenotypically resistant *Culex* spp. in an urban locality (Bioko Island) of Equatorial Guinea between 2014 and 2017 to four major classes of insecticides (pyrethroid, organochlorine, carbamate, organophosphate) associated with an indoor residual spraying (IRS) campaign. During the same period, biting rates of less resistant *Anopheles gambiae* s.l. remained steady [32]. Thymol selected mosquitoes, however, had a high biting rate at both doses, suggesting that thresholds for this chemical to affect avidity are lower.

### Fecundity

We found an overall increase in the number of eggs in the first gonotrophic cycle associated with phenotypic resistance to both permethrin and thymol in *Ae. aegypti*, with higher fecundity being most prominent among mosquitoes selected at the strongest selection pressure (i.e., high doses).

Permethrin. Selection for resistance to permethrin in mosquitoes is known to impact fecundity [27,33]. Results from the current study revealed *Ae. aegypti* female selection at the high dose (120 ug) of permethrin laid significantly more eggs compared to the control parental population. Increased fecundity in the selected population could mean that resistant mosquitoes could outcompete susceptible *Ae. aegypti* in nature although our studies revealed permethrin selection also decreased hatch rate. These results, in combination with compromised longevity of resistant individuals, may suggest energy reserves compensating for a trade-off between permethrin resistance and life history traits, as the maintenance of metabolic resistance (overexpression of detoxification enzymes) and target site mechanisms can be costly and require reallocation of energetic resources in insects [34]. For example, pyrethroid resistant *An. gambiae* had compromised reproduction (e.g., fecundity, duration of gonotrophic cycle, and net reproduction rate) but greater longevity relative to susceptible conspecifics from the same geographic area [35]. Differences in patterns of life history trait trade-offs among resistant insects (higher or lower fecundity and longevity) may, in part, be attributable to physiological decisions on use of stored lipids for detoxification or reproduction (e.g., planthopper *Nilaparvata lugens* [36]. However, we only determined fecundity for the first gonotrophic cycle, which may not be representative of lifetime fecundity. The consequence of enhanced fecundity should be assessed in context of associated reductions in viability of eggs and longevity of adults, which are likely to offset the fitness enhancement. The widespread use of pyrethroids and resistance in *Ae. aegypti* populations around the world is consistent with the hypothetical situation of fitness enhancement. The increase in fecundity in the laboratory selected permethrin resistant *Ae. aegypti* could be due to selection of resistance in both the males and females. Progeny from matings are likely very robust compared to parental mosquitoes. Some of the observed results (lower hatch rates of high dose selected line) suggest that permethrin selection impeded egg development. Upon review of the literature, the impact of permethrin resistance on fecundity has shown mixed results. An assessment of changes in life characteristics in a related species, *Ae. albopictus,* following 10 generations of selection to permethrin resulted in a 50% reduction in hatchability of eggs and tended to have longer development among the immature stages compared to their susceptible counterparts [37]. The authors suggested that phenotypically resistant individuals may be redirecting nutrient resources into enhancement of detoxifying enzymes (monooxygenase, β-esterase, Glutathione S-transferases) at the cost of diminished life history attributes. Field *Ae. aegypti* collected in different states in Brazil and selected in the laboratory for resistance to deltamethrin laid fewer eggs than the unselected control population [28]. However, the fecundity of deltamethrin resistant populations was compared with a long-standing laboratory colony and thus interpretation of these results should be done with caution. Gleave et al. [33] collected *Ae. aegypti* from Recife and selected for resistance to temephos, permethrin and malathion. The results showed selection with permethrin increased egg laying compared to unselected females, but the difference was not significant. The mean number of eggs was higher for Australian populations of *Ae. aegypti* highly resistant to permethrin, and with additional resistance to lambda-cyhalothrin and deltamethrin, compared to the control population [29]. However, these mosquitoes were resistant to multiple chemicals and thus development of resistance and cross-resistance between insecticides could have added effects on fecundity.

Thymol. Selection for resistance to the thyme essential oil component, thymol, increased fecundity in the first gonotrophic cycle at both doses, and egg numbers were significantly different compared to the control Fxx4 population of *Ae. aegypti*. This is the first study to suggest that insensitivity to an essential oil can develop from repeated generational exposure and associated selection. Additionally, this is the first study to link insensitivity to an essential oil component to increased fecundity and viability of eggs; the combination of both effects can compromise control efforts. Studies about the impact of essential oils and their components on egg laying have exclusively concentrated on oviposition deterrence [14,38,39]. Thymol, the major component of the seed of the plant *Tr*. *ammi*, and the essential oil from the seeds significantly deterred oviposition for *An. stephensi*. Additionally, eggs laid in solutions containing both chemicals were less viable [14]. It is unknown whether selection for insensitivity to thymol would affect oviposition deterrence from larval habitats containing these chemicals.

### Adult development

We show that permethrin resistance in mosquitoes may shorten development time resulting in fitness advantages over susceptible populations. This scenario will impact mosquito control efforts supporting the need for alternative control measures, such as use of essential oils. We found that selection with thymol delays emergence of adults, but the significance is not clear.

Permethrin. Selection with permethrin resulted in sex and dose specific differences in development. Development time for both male and female *Ae. aegypti* selected at the low dose of permethrin was shorter compared to parental control population. Male mosquitoes selected with the high dose had similar development times as the control mosquitoes, however, female *Ae. aegypti* selected at the high dose had faster development compared to the control Fxx4 population. These results suggest that permethrin selection shortened development time, regardless of dose, suggesting a fitness advantage. Additionally, sex-dependent outcomes are likely attributable to sexually dimorphic development time, growth rate, and size in *Ae. aegypti*, because adult male mosquitoes are usually smaller than females and emerge to adulthood sooner [40]. Gleave et al. [33] investigated eclosion for laboratory selected permethrin resistant mosquitoes and found shorter development time for males compared to female *Ae. aegypti* but these data were not significantly different from the unselected parental population.

Thymol. Thymol selection slowed *Ae. aegypti* development to adults compared to the parental control population and the delay exhibited a dose-dependent relationship. This observation suggests that selection with an essential oil component influences adult emergence. Although this study suggests that selection with thymol increases fecundity compared to control *Ae. aegypti*, a lag in adult development might counter any advantage conferred by egg number. Studies about the influence of essential oil or an active ingredient on mosquito development are lacking. However, studies on hatchability and development of eggs exposed to essential oils generally showed high larval mortality thus estimates of temporal progression of development were not possible [14,38,39]. Pandey et al. [14] showed lower hatch rate for *An. stephensi* eggs laid in oviposition media containing *Tr. ammi* or its major component, thymol, and higher doses of both chemicals resulted in high larval mortality. As with other life history traits, though, the effects of direct exposure on an insensitive population are not yet known.

Faster development and consequent shorter generation time can lead to increased size of populations, but may also result in mosquitoes with smaller body sizes. Changes in body size can affect fecundity, vector competence, and other mosquito traits, but can be difficult to separate from temperature and nutrition effects. The net effects of faster development on population size and vectorial capacity can be difficult to assess.

Taken together, the fecundity and initial feeding propensity suggests that resistance to permethrin and thymol may confer a positive advantage to mosquito populations. However, the longevity studies suggest that higher resistance impacts longevity and there is an interaction with longevity and mating. Additionally, further studies are needed to assess the influence of insecticide resistance or insensitivity to repellents on other parameters relevant to the ability of *Ae. aegypti* mosquitoes to transmit arboviruses, such as the extrinsic incubation period [41]. Selected populations from this current study have been used to assess vector competence for dengue −1 virus. A manuscript discussing the influence of permethrin and thymol resistance on DENV-1 infection, dissemination and transmission rate will be published separately.

## Supporting information

S1 Fig Flow chart of mosquito selection scheme for permethrin and thymol. (TIF)

S2 Fig Life history experiments flow chart. (TIF)

S3 Fig Choice chamber used for thymol selection. (TIF)

S1 Table Sample sizes for life history studies. (XLSX)

S2 Table Means comparisons for biting rate experiment. (XLSX)

S3 Table Means comparisons for fecundity estimation. A, ANOVA for fecundity; B, means comparisons for fecundity; C, *Χ*^2^ Sq for fecundity proportion egg laying; D, *Χ*^2^ Sq fertility proportion egg hatch.
(XLSX)

S4 Table Development rate statistics. A, ANOVA of permethrin-selected male population; B, means comparisons for permethrin-selected male population; C, means comparisons for permethrin-selected female population (ANOVA results in main text); D, ANOVA of thymol-selected male population; E, means comparisons for thymol-selected male population; F, means comparisons for thymol-selected female population (ANOVA results in main text).
(XLSX)

S5 Table Emergence statistics, Kruskal-Wallis tests. A, permethrin selected lines; B, thymol selected lines; C, means comparison Thymol selected lines.
(XLSX)

S6 Table Proportion female statistics, Kruskal-Wallis tests. A, permethrin selected lines; B, thymol selected lines.
(XLSX)
