# Supplementary figures and images for: Changes in the life history traits in Aedes aegypti selected for resistance to permethrin and thymol, a spatial repellent
Source: PLoS One. 2025 Aug 19;20(8):e0329776. doi: 10.1371/journal.pone.0329776 (PMC12364310; doi:10.1371/journal.pone.0329776)

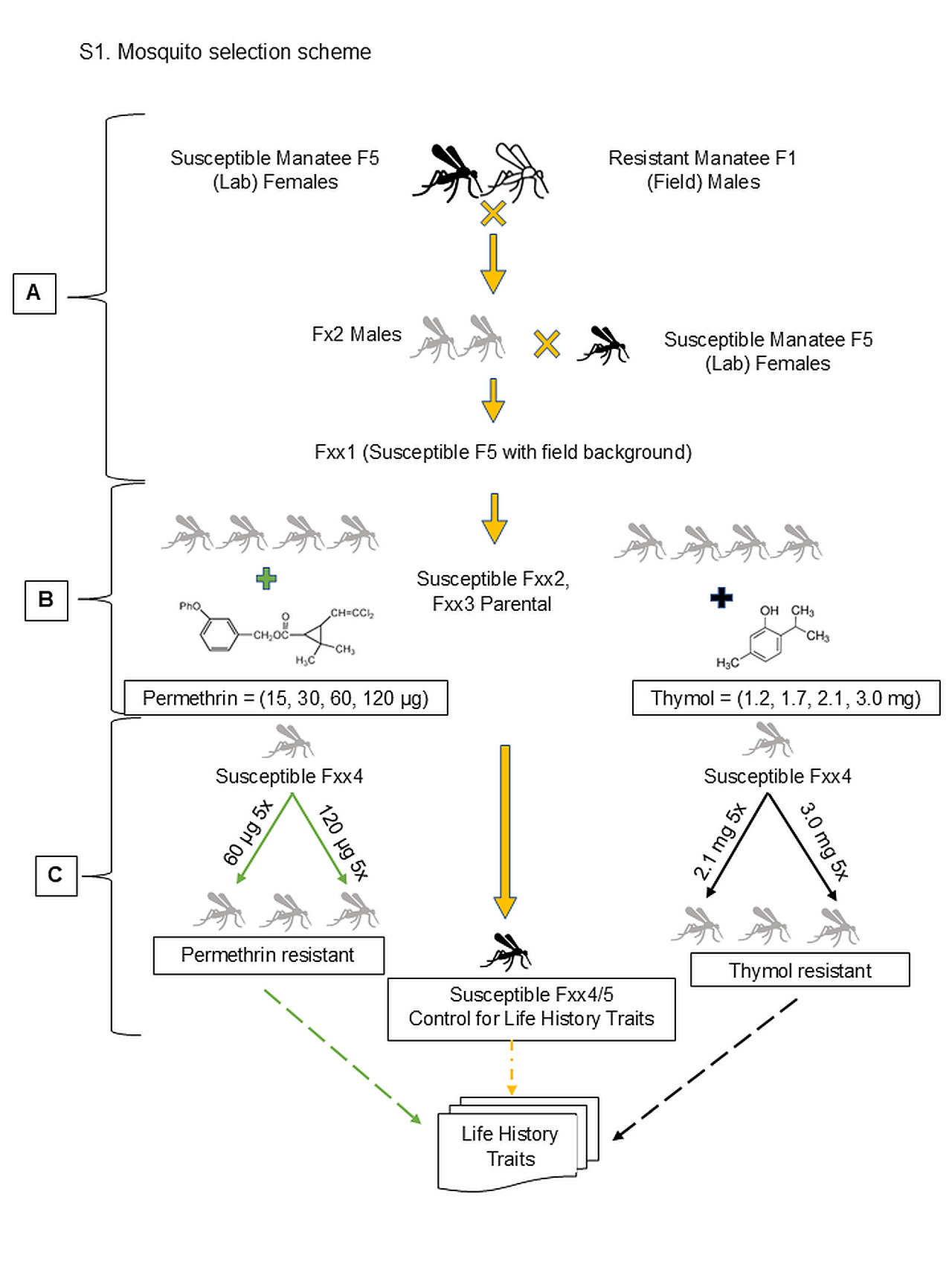

Supplement: S1 Fig — (TIF) [file pone.0329776.s001.tif]

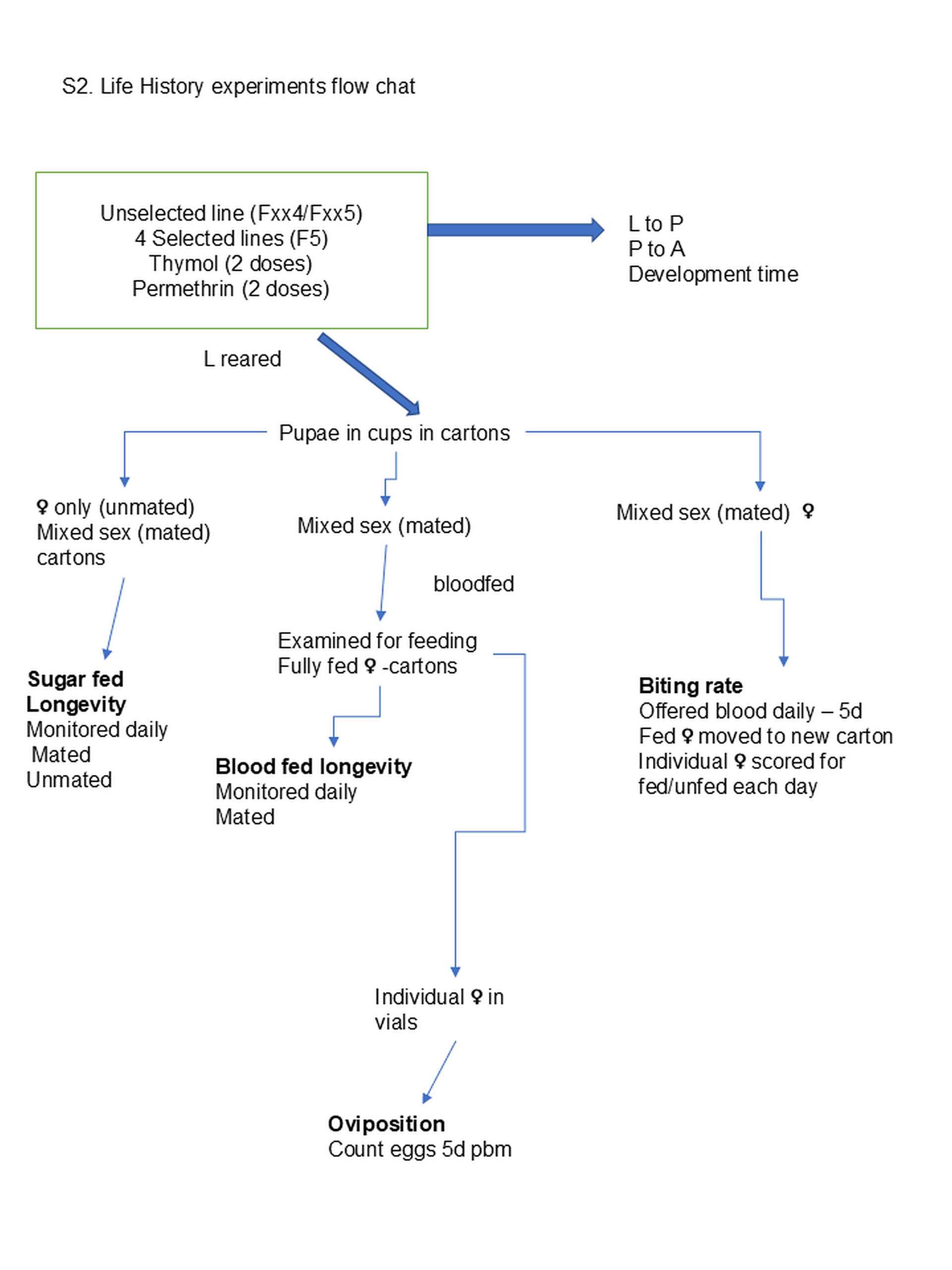

Supplement: S2 Fig — (TIF) [file pone.0329776.s002.tif]

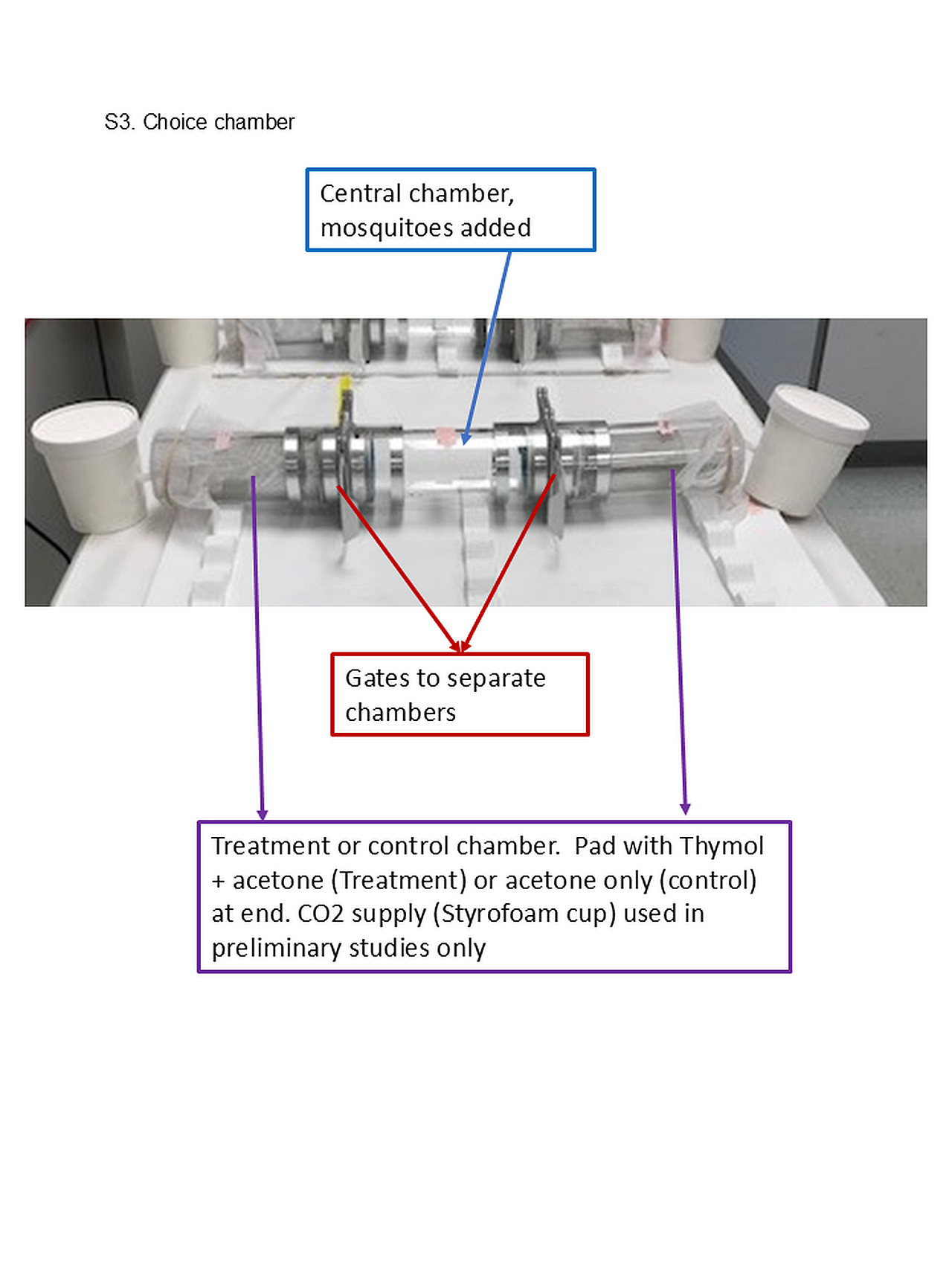

Supplement: S3 Fig — (TIF) [file pone.0329776.s003.tif]
